# Bidirectional associations between maternal and paternal depressive symptoms across the postpartum period: Contributions of maternal prenatal depression and childhood neglect

**DOI:** 10.1017/S0954579426101576

**Published:** 2026-06-11

**Authors:** Megan Hare, Kathryn Humphreys

**Affiliations:** 1 Department of Psychology and Human Development, Peabody College, Vanderbilt University, USA; 2 Department of Psychiatry and Behavioral Sciences, Tulane University, USA

**Keywords:** depression, fathers, mothers, neglect, peripartum

## Abstract

Postpartum depression (PPD) is a major public health problem. Although maternal PPD has received more attention, less is known about paternal PPD and how parents’ depressive symptoms influence one another over time. The present study investigated longitudinal patterns of maternal and paternal depressive symptoms during the first 18 months postpartum using a random intercept cross-lagged panel model in 119 mothers (M_age_ = 31.15, 89% White, 93% non-Hispanic, 91% married) and fathers (M_age_ = 32.74), while also considering the role of maternal prenatal depression and maternal childhood neglect. Results indicated strong autoregressive stability for maternal depressive symptoms across all time points (*β* = 0.24–0.61), whereas paternal symptoms showed less consistency, with stability emerging later. Our results revealed a bidirectional effect, where maternal symptoms at 1 month (*β* = 0.35) predicted paternal symptoms at 6 months (*β* = 0.26), which in turn predicted maternal symptoms at 12 months (*β* = 0.28). Additionally maternal emotional (*β* = 0.33) and physical neglect (*β* = 0.18) were associated with higher maternal depressive symptoms during pregnancy, and emotional neglect (*β* = 0.35), uniquely predicted higher maternal depressive symptoms at 1 month postpartum. These findings highlight the interconnected nature of parental depressive symptoms and the lasting impact of maternal neglect on early postpartum mental health.

Postpartum depression (PPD) is a significant public health concern with substantial implications for parental well-being, infant development, and family functioning. PPD is currently classified in the Diagnostic and Statistical Manual of Mental Disorders (DSM-5) as a specifier for major depressive disorder (i.e., MDD with peripartum onset; American Psychiatric Association, 2022) and includes core symptoms of MDD, such as persistent sadness, loss of interest or pleasure, changes in appetite or sleep, fatigue, feelings of worthlessness or guilt, and difficulty concentrating. However, PPD can also present with features that are uniquely salient to the perinatal context, including increased anxiety, intrusive thoughts related to infant harm, irritability, heightened sensitivity to stress, and difficulty bonding with the infant (O’Hara & Wisner, 2014; Shorey et al., 2018). Further, PPD is well documented as a risk factor for disrupted mother–infant bonding, lower maternal sensitivity, and increased risk for child developmental difficulties, including emotional and behavioral dysregulation (Goodman, 2004; Goodman et al., 2022; La Rosa et al., 2025). Prevalence estimates for PPD in mothers range from 10%–25% (Shorey et al., 2018; Wang et al., 2021a; Woody et al., 2017), with higher rates reported in studies using self-report screening tools or in populations experiencing elevated psychosocial stress (Hahn-Holbrook et al., 2018; Khadka et al., 2024). Although maternal PPD has received increasing attention in both research and clinical settings, it remains underdiagnosed and undertreated, with many affected individuals not receiving adequate care (Bauman, 2020) despite consequences for both mother and child. Untreated PPD in mothers can lead to chronic depression, reduced quality of life, and an increased risk of future mental health disorders (Biaggi et al., 2016; Stewart & Vigod, 2019).

Paternal PPD, though less frequently studied, is also a prevalent concern, affecting between 8%–25% of fathers (Álvarez-García et al., 2024; Cameron et al., 2016; Smythe et al., 2022). Similar to maternal PPD, paternal PPD often goes undiagnosed and undertreated, as screening and treatment efforts rarely target fathers (Papaleontiou - Louca & Al Omari, 2020), despite evidence that fathers’ mental health plays a critical role in family dynamics and child development. Like maternal depression, paternal PPD has been linked to adverse outcomes, including lower relationship satisfaction, increased parenting stress, and elevated risk for substance use and other mental health concerns (Schmitz, 2025; Shorey & Chan, 2020; Suto et al., 2016). Furthermore, paternal PPD is associated with reduced engagement in caregiving, harsher parenting practices, and a higher likelihood of child externalizing behaviors (Sethna et al., 2018; Sweeney & MacBeth, 2016; Wilson & Durbin, 2010). These parallels highlight the importance of considering both maternal and paternal mental health when assessing PPD and its long-term consequences for families.

These adverse consequences are not limited to individual parents; rather, they can reverberate throughout the family system. In particular, depressive symptoms in one parent can affect the other, potentially creating a cycle of distress within the couple and amplifying risk for the child (Cummings & Davies, 2011; Williams & Cheadle, 2015). Prior findings suggest that maternal PPD is among the strongest predictors of paternal PPD (Goodman, 2004; Wee et al., 2013), with evidence indicating that fathers experience elevated depressive symptoms, sometimes reaching clinical levels in up to 50% of cases, when their partners also experience PPD (Shorey & Chan, 2020). Conversely, paternal depression has also been found to be associated with increases in maternal depressive symptoms over time (Paulson & Bazemore, 2010). In addition to interpersonal and contextual mechanisms, associations between maternal and paternal depressive symptoms may partly reflect selection processes, such as assortative mating, whereby individuals with similar vulnerabilities to depression are more likely to partner with one another (Mathews & Reus, 2001). Such selection effects may contribute to baseline concordance in depressive symptoms within couples, particularly during periods of heightened stress. These bidirectional associations between maternal and paternal depression may stem from increased caregiving demands, relational distress, or shifts in family dynamics. The transition to parenthood is also a period of heightened vulnerability to mental health difficulties, with evolving roles, responsibilities, and sleep disruptions contributing to psychological distress in both parents (Mckenzie & Carter, 2013; Philpott et al., 2017). Importantly, selection-based processes and stress-based spillover mechanisms are not mutually exclusive. Preexisting vulnerabilities within couples may interact with the acute demands of pregnancy and early parenthood, creating conditions under which depressive symptoms become mutually reinforcing over time. Given that parents’ mental health is mutually influential, addressing depression in one parent without considering its impact on the other may limit the effectiveness of interventions. Therefore, a more comprehensive approach that accounts for the dyadic and family-level context of PPD is essential for improving outcomes for both parents and their children.

The bidirectional association between maternal and paternal depression is supported by family systems theory and the stress spillover model, which highlight how partners’ emotional and behavioral functioning are deeply interconnected within the family. Although there is growing awareness of the mutual influence between maternal and paternal depressive symptoms, research on their reciprocal effects over time remains limited. Much of the existing work relies on cross-sectional designs (Anding et al., 2016; Matthey et al., 2000; Wang et al., 2021b), unidirectional models focusing on only one direction of influence (Paulson et al., 2016; Roubinov et al., 2014), or separate analyses of maternal and paternal depression rather than examining their longitudinal interdependence (Kiviruusu et al., 2020; Sipsma et al., 2016).

Additionally, studies exploring bidirectional effects often use between-person comparisons, which demonstrate general associations but do not reveal how changes in one parent’s depressive symptoms over time may drive corresponding changes in their partner’s symptoms. Between-person effects capture whether parents who are more depressed than other parents tend to have partners who are also more depressed; these associations reflect stable differences between families and cannot determine whether changes in one parent’s symptoms predict changes in the other’s symptoms over time. In contrast, within-person effects capture whether a parent experiences increases or decreases in depressive symptoms relative to their own typical level, and whether these time-specific fluctuations forecast subsequent changes in their partner’s symptoms. This distinction is critical in dyadic research, as between-dyad differences may reflect stable characteristics of couples, whereas within-dyad processes reveal how partners dynamically influence each other over time. As a result, the extent to which fluctuations in one parent’s symptoms influence those of the other remains unclear.

Recent advancements in statistical modeling have provided new opportunities to disentangle the dynamic interplay of depressive symptoms between parents. One such approach, the random intercept cross-lagged panel model (RI-CLPM), offers a more precise method for examining within-person and between-person effects over time. Clifford et al. (2024) were the first to apply this model to examine the bidirectional influences of maternal and paternal depressive symptoms measured once per year from 6 to 54 months postpartum. Their analyses revealed a unidirectional effect, in which maternal depression was associated with paternal depressive symptoms across two intervals (18 to 30 months and 30 to 42 months postpartum), but the reverse pattern was not found. Additionally, mothers who exhibited higher-than-usual depressive symptoms at one time point were more likely to report elevated symptoms at subsequent assessments throughout the study period, suggesting a self-reinforcing pattern of maternal depression over time. In contrast, paternal depressive symptoms showed a less consistent influence on later paternal symptoms, with only one statistically significant interval identified.

While Clifford et al. (2024) made important contributions to understanding the dynamics of parental depression, their design may have missed critical processes unfolding earlier in the perinatal period, including during pregnancy and the immediate postpartum months. Depressive symptoms frequently emerge during pregnancy, and prenatal depression is among the strongest predictors of PPD for mothers (Biaggi et al., 2016; Stewart & Vigod, 2019). Pregnancy itself represents a period of heightened vulnerability, marked by physiological changes, anticipatory stress, and shifts in relational expectations that may initiate or amplify dyadic emotional processes even before the infant’s arrival. Capturing symptoms in the immediate postpartum period is also critical, as this window represents a particularly vulnerable time for both parents, marked by rapid hormonal changes, sleep disruption, and the onset of new caregiving demands (Suryawanshi & Pajai, 2022), all of which can shape depressive symptoms and influence parent–infant bonding and family functioning (La Rosa et al., 2025). Furthermore, there is considerable variability in when depressive symptoms first emerge. For instance, some mothers experience early-onset or chronic depression, while others show delayed onset, with symptoms arising between 6 and 36 months postpartum (Netsi et al., 2018; Wang et al., 2021a). Paternal depression may follow yet another pattern, often emerging later and persisting longer than maternal depression (Bruno et al., 2020; Wee et al., 2013).

Perinatal depressive symptoms (i.e., depressive symptoms occurring during pregnancy through the first year postpartum) are often dynamic rather than static, with fluctuations in severity that may carry meaningful implications for parenting and family functioning. Family systems theory and stress spillover models propose that emotional influence between partners occurs over relatively short timescales, as shifts in one partner’s mood, stress, or coping capacity can rapidly shape the emotional climate of the couple (Almeida et al., 1999; Cox & Paley, 1997). From this perspective, prenatal depressive symptoms may represent an early entry point into dyadic cascades of risk, setting the stage for how parents respond to postpartum challenges and to each other. However, widely spaced assessment intervals may limit the ability to detect such short-term reciprocal processes, highlighting the need for more frequent assessments that can capture dynamic changes in depressive symptoms as they unfold across the perinatal period.

Beyond understanding dyadic bidirectional effects, identifying developmental and intergenerational risk factors that shape the onset of and vulnerability to perinatal depression is essential. One such factor is parents’ own early caregiving experiences. Substantial research indicates that childhood maltreatment confers risk for prenatal and postpartum mental health difficulties, in part through heightened parenting stress and impaired bonding (Ayers et al., 2019; Frohberg et al., 2022; Souch et al., 2022), both of which are associated with increased risk for perinatal depression (Lehnig et al., 2019). For instance, Lehnig et al. (2019) found that maternal childhood maltreatment was linked to more negative feelings about the mother–infant relationship, which in turn predicted higher levels of postpartum depressive symptoms. While prior work has established links between childhood maltreatment and maternal prenatal and postpartum depressive symptoms (e.g., Hutchens et al., 2017; Madigan et al., 2015) much less is known about how specific types of maltreatment influence perinatal depression risk. Childhood neglect is the most prevalent form of maltreatment (U.S. Department of Health & Human Services, 2023), yet its unique developmental consequences remain insufficiently understood.

Neglect is particularly relevant to prenatal depressive symptoms, which often mark the onset of perinatal depression. Emotional neglect, characterized by the absence of emotional responsiveness, validation, and support, has been shown to disrupt the development of emotion awareness, emotion labeling, and adaptive emotion regulation strategies from childhood into adulthood (Greene et al., 2021; Kim & Cicchetti, 2010; Pollak & Sinha, 2002; Shipman & Zeman, 2001). These disruptions are associated with heightened stress sensitivity, difficulty identifying and tolerating affective states, and reliance on maladaptive coping, processes that represent well-established vulnerability factors for depression during pregnancy (Simon et al., 2024). Pregnancy is also a period of substantial physiological, emotional, and relational change, during which emotions relevant to early caregiving experiences may become reactivated, amplifying these vulnerabilities. In contrast, physical neglect, characterized by chronic exposure to unmet basic needs and environmental instability, has been linked to long-term alterations in stress physiology, including dysregulation of the hypothalamic–pituitary–adrenal (HPA) axis (Danese & McEwen, 2012; Gunnar & Quevedo, 2007). During pregnancy, when physical demands, health concerns, and stress exposure often intensify, these stress-related vulnerabilities may be particularly salient.

Neglect-related vulnerabilities that emerge during pregnancy may also extend into the postpartum period, when ongoing sleep disruption, heightened caregiving demands, and rapid role transitions can maintain or exacerbate depressive symptoms (Souch et al., 2022; Stewart & Vigod, 2019). Thus, maternal childhood neglect may influence PPD both directly and indirectly via prenatal depressive symptoms. Consistent with these mechanisms, studies that include neglect within broader maltreatment or adversity composites demonstrate that childhood adversity predicts higher prenatal depressive symptoms and increased overall risk for perinatal psychopathology (Choi & Sikkema, 2016; Racine et al., 2020; Wajid et al., 2020). However, because neglect is typically embedded within composite maltreatment indices, it remains unclear whether emotional and physical neglect confer risk through shared or distinct pathways, and whether one form is more strongly implicated in the emergence of prenatal depressive symptoms. Despite this strong theoretical rationale, no studies to date have systematically examined whether emotional and physical neglect show differential associations with prenatal depression, early PPD, or changes in symptoms over time.

Moreover, from a family systems and stress spillover perspective (Almeida et al., 1999; Cox & Paley, 1997), maternal neglect may also have implications for paternal adjustment. While no studies have directly tested whether maternal childhood neglect predicts paternal PPD, neglect-related difficulties in mothers, such as elevated prenatal and PPD, impaired emotion regulation, and heightened stress reactivity, may indirectly shape fathers’ symptoms by increasing relational strain, coparenting difficulties, and everyday emotional spillover (Almeida et al., 1999; Williams & Cheadle, 2016). Thus, examining whether maternal childhood neglect indirectly predicts fathers’ early PPD via maternal perinatal symptoms is theoretically grounded and aligns with established dyadic models of family functioning.

The current study examined longitudinal patterns of maternal and paternal depressive symptoms across the first 18 months postpartum (1, 6, 12, and 18 months) using a RI-CLPM. Guided by family systems theory, stress spillover models, and developmental psychopathology frameworks, the study addressed three primary aims. Aim 1 was to characterize within-person stability and bidirectional within-dyad spillover of maternal and paternal depressive symptoms across the postpartum period. Consistent with prior work, we hypothesized moderate to strong stability in maternal depressive symptoms (Clifford et al., 2024; Jacques et al., 2020), reflecting continuity once symptoms emerge, and weaker stability in paternal depressive symptoms between 1 and 6 months postpartum, with stability emerging between 6 and 18 months postpartum (Bruno et al., 2020; Paulson & Bazemore, 2010).

We further hypothesized reciprocal spillover effects such that increases in one parent’s depressive symptoms relative to their own typical level would predict subsequent increases in their partner’s symptoms over time (Almeida et al., 1999; Cox & Paley, 1997). Specifically, we hypothesized that maternal depressive symptoms at 1 month postpartum would predict increases in paternal depressive symptoms at 6 months postpartum, given mothers’ central role in early infant care and evidence that maternal distress strongly shapes the early family emotional climate (Goodman, 2004; Shorey & Chan, 2020). In turn, we hypothesized that paternal depressive symptoms at 6 or 12 months postpartum would predict subsequent increases in maternal depressive symptoms at the following assessment (12 or 18 months postpartum), reflecting accumulating caregiving demands, coparenting strain, and reciprocal stress processes as family roles stabilize over time (Paulson & Bazemore, 2010; Williams & Cheadle, 2015).

Aim 2 was to examine the role of maternal prenatal depressive symptoms in shaping early postpartum adjustment for both parents. We hypothesized that maternal prenatal depressive symptoms would predict higher maternal depressive symptoms at 1 month postpartum, including elevations relative to mothers’ typical postpartum symptom level, consistent with extensive evidence that PPD often represents a continuation of symptoms that emerge during pregnancy (O’Hara & Wisner, 2014; Underwood et al., 2016).

Aim 3 was to examine whether maternal childhood emotional and physical neglect conferred vulnerability to maternal perinatal depressive symptoms. We tested whether maternal neglect was associated with higher depressive symptoms during pregnancy and higher depressive symptoms at 1 month postpartum. We hypothesized that both emotional and physical neglect would be associated with elevated prenatal depressive symptoms (Monk et al., 2019; Repetti & Bai, 2025), a period of heightened biological and psychological sensitivity during which early caregiving experiences may become reactivated. We further hypothesized that emotional neglect would be uniquely associated with higher maternal depressive symptoms at 1 month postpartum, given evidence that emotional neglect disrupts emotion awareness, self-soothing, and support-seeking, processes that are especially salient during the immediate postpartum period (Kim & Cicchetti, 2010; Monk et al., 2019).

In addition, we hypothesized that maternal childhood neglect would not directly predict paternal depressive symptoms at 1 month postpartum but would be indirectly associated with paternal symptoms via elevated maternal prenatal and early postpartum depressive symptoms. This hypothesis was grounded in family systems and stress spillover models, which suggest that early caregiving adversity influences partner adjustment primarily through its impact on maternal emotional functioning rather than through direct cross-parent effects (Almeida et al., 1999; Williams & Cheadle, 2015).

## Methods

### Participants

Participants were drawn from a longitudinal study investigating associations between early experiences and brain development in infancy. Pregnant women were recruited from a large metropolitan city in the central southeastern part of the United States. In total, 328 participants were enrolled in the study and provided data during pregnancy. Although the original study design focused on one primary caregiver, partners were invited to participate beginning at the 1 month postpartum assessment and could enroll in the study at any postpartum time point. All participants in the current sample identified as women, while all secondary caregivers identified as men; therefore, we will refer to them as mothers and fathers, respectively. Almost all mothers reported being married or in a domestic partnership (91%), with 6% reporting being single and never married, 2% divorced, and 1% separated. In addition, most mothers reported being White (89%), and non-Hispanic (93%), with a mean age of 31.15 (*SD* = 4.30) at the time of the pregnancy assessment. More than half of mothers (64%) reported that this was their first pregnancy. Fathers had a mean age of 32.74 (*SD* = 5.54) at the time of the pregnancy assessment. All but two fathers reported currently living with the child’s mother; one father reported not living with the child’s mother, and one did not provide information on cohabitation status. See Table 1 for more detailed sample characteristics.

### Procedures

All procedures were approved by Vanderbilt University Institutional ReviewBoard. All recruitment and studyproceduresperformed were in accordance with the prevailing ethical standards. Participants provided informed consent before completing questionnaires. Participants were recruited during pregnancy from local obstetric clinics, printed advertisements, and social media advertising. Participants completed multiple assessments, including batteries of online questionnaires during pregnancy and at 1, 6, 12, and 18 months postpartum. Data were collected and managed using REDCap (Garcia & Abrahão, 2021). Participants received a $20 to $40 gift card, depending on time point, for participation. Only birthing parents participated during pregnancy, but in two-parent households, both parents were invited to participate in all postnatal data collection waves if contact information was provided. Eligibility requirements for participants included (a) U.S. citizenship or permanent residence, (b) 18 years of age or older, (c) fluency in English, and (d) currently pregnant with a singleton pregnancy, between 11- and 38-weeks’ gestation.

### Measures

#### Parental depression

The Center for Epidemiologic Studies Depression Scale (CES-D) (Radloff, 1977) was used to assess depressive symptoms prenatally (mothers only) and at 1-, 6-, 12- and 18-months postpartum for both parents. The CES-D is a widely used self-report measure designed to capture the frequency of depressive symptoms experienced over the past week. It consists of 20 items that assess various aspects of depression, including mood, appetite, sleep, and energy levels, as well as feelings of guilt, worthlessness, and thoughts of death. Participants rate each item on a 4-point scale, ranging from “rarely or none of the time” to “most or all of the time.” The CES-D has previously demonstrated strong psychometric properties (Lewinsohn et al., 1997; Vilagut et al., 2016) and had high internal consistency within the current sample (*α*-values ranged from 0.76–0.90 in mothers and 0.88–0.91 in fathers). Clinically elevated depressive symptoms were defined using the standard cutoff score of 16 or higher, which has been widely used to indicate risk for clinically significant depressive symptomatology in perinatal samples (Radloff, 1977; Weissman et al., 1977). Across the perinatal period, the proportion of mothers scoring above the clinical cutoff for depressive symptoms on the CES-D was 7% prenatally, 28% at 1 month postpartum, 12% at 6 months, 11% at 12 months, and 13% at 18 months. For fathers, the proportion scoring above the clinical cutoff for depressive symptoms on the CES-D was 13% at 1 month postpartum, 11% at 6 months, 13% at 12 months, and 9% at 18 months.

#### Maternal history of neglect

Maternal history of childhood neglect was assessed during pregnancy using the Childhood Trauma Questionnaire–Short Form (CTQ-SF) (Bernstein et al., 2003), a widely used 28-item retrospective self-report measure of maltreatment experiences before age 18. The CTQ yields five subscales: Emotional Neglect, Physical Neglect, Emotional Abuse, Physical Abuse, and Sexual Abuse. Only the two neglect subscales, Emotional Neglect and Physical Neglect, were used in the present study. The Emotional Neglect subscale (5 items) assesses the extent to which caregivers failed to provide emotional support, warmth, and affection (e.g., “There was someone in my family who helped me feel important or special,” reverse scored). The Physical Neglect subscale (5 items) captures the lack of basic physical care or safety (e.g., “I didn’t have enough to eat”). Mothers rated each item on a 5-point Likert scale (1 = *Never True* to 5 = *Very Often True*), with higher scores indicating greater severity of neglect. Subscale scores were computed by summing items within each domain. The CTQ was administered only to mothers; fathers did not provide retrospective maltreatment histories.

### Analytic plan

#### Missing data

Although 328 birthing parents were enrolled prenatally, participation of secondary caregivers (typically fathers) was optional and began at the 1 month postpartum assessment. As a result, the dyadic analytic sample reflects the subset of families in which both parents opted into participation and provided sufficient repeated measures for longitudinal dyadic modeling. In total, 322 mothers completed the CTQ and CES-D at the prenatal time point, 245 mothers and 140 fathers provided CES-D data at 1 month, 195 mothers and 86 fathers provided data at 6 months, 118 mothers and 76 fathers provided data at 12 months, and 161 mothers and 86 fathers provided data at 18 months. The 12-month assessment was introduced later in the study, contributing to lower participation at that wave. The RI-CLPM requires at least two repeated observations per individual to estimate stable between-person random intercepts and time-varying within-person latent components. Although full information maximum likelihood (FIML) effectively handles missing repeated-measures data, individuals with only a single observation cannot meaningfully contribute to within-person or cross-lagged estimates and would result in model underidentification (Hamaker et al., 2015; Mulder & Hamaker, 2021). Therefore, analyses were restricted to dyads in which both parents completed at least two depression assessments, yielding a final sample of 119 dyads.

Patterns of missing data were examined using Little’s MCAR test, which was non-significant, *χ*^2^(178) = 79.00, *p* = .170, suggesting that missingness was not systematically related to observed variables. Families included versus excluded from the analytic sample did not differ on key sociodemographic characteristics (i.e., biological sex, race, income, child biological sex, maternal neglect, or baseline depressive symptoms; *p*s > .05). Models were therefore estimated using FIML. Simulation work using the *powRICLPM* R package suggests that samples of approximately 100–200 individuals with 3–5 waves are powered to detect moderate within-person and cross-lagged effects, but are underpowered for very small effects (Mulder, 2023). The present analyses included 238 individuals (119 dyads) assessed across four waves, placing the study within the range expected to detect moderate dyadic spillover effects. Accordingly, nonsignificant paths, especially those with very small point estimates, should be interpreted with caution.

We calculated the intraclass correlation coefficients (ICCs) for the repeated assessments of maternal and paternal depressive symptoms to quantify the stability of depressive symptoms and determine the appropriateness of using a RI-CLPM. In this context, the ICC indicates the proportion of the total variance in depressive symptoms that is attributable to differences between individuals across assessments, with higher values indicating greater stability in symptoms over time and lower values indicating more within-person variability. The ICCs therefore help determine whether a RI-CLPM is justified: the model is inappropriate if ICCs are extremely high (indicating almost no within-person fluctuation to model) or extremely low (suggesting that a model incorporating growth trajectories or alternative forms of change may be more appropriate).

We employed a RI-CLPM in MPlus 8.1 (Muthén & Muthén, 2017) to examine the reciprocal associations between maternal and paternal depressive symptoms over time while accounting for individual variability. Unlike traditional cross-lagged models, the RI-CLPM separates stable, trait-like differences between individuals from time-specific fluctuations, allowing for a more precise examination of within-person changes. We selected the RI-CLPM over alternative longitudinal models (e.g., autoregressive latent trajectory model with structured residuals [ALT-SR], and a latent curve model with structured residuals [LCM-SR]) because the primary research question focused on time-anchored within-person and within-dyad processes rather than on estimating average developmental trajectories. Given that depressive symptoms can vary meaningfully across the postpartum period even in the absence of systematic linear or nonlinear change, the RI-CLPM provided a parsimonious and theoretically appropriate framework for isolating dynamic dyadic associations.

To evaluate whether systematic between-person variability in depressive symptom trajectories influenced model selection, we also estimated an alternative longitudinal model using a LCM-SR (Curran et al., 2014). This model allows simultaneous estimation of individual differences in growth trajectories (intercepts and slopes) while modeling autoregressive relations among time-specific residuals. The LCM-SR was estimated using MLR and FIML. Model convergence and parameter estimates were examined to determine whether reliable slope variability could be estimated in the present sample.

We then extended the RI-CLPM to incorporate maternal emotional neglect, physical neglect, and prenatal depressive symptoms as time-invariant predictors (Mulder & Hamaker, 2021). Specifically, maternal emotional and physical neglect were specified as predictors of (a) maternal prenatal depressive symptoms, (b) the random intercepts for maternal and paternal depressive symptoms, representing stable between-person differences across the postpartum period, and (c) the latent within-person deviation factors at 1 month postpartum for both parents, indexing early postpartum symptom elevations relative to each parent’s expected level after accounting for stable differences. Maternal prenatal depressive symptoms were specified analogously, predicting the maternal and paternal random intercepts, as well as the 1 month postpartum within-person deviations for both parents. This specification allowed us to distinguish stable, trait-like vulnerability associated with early adversity and prenatal depression from their associations with early postpartum symptom elevations, while preserving the interpretation of later cross-lagged paths as purely within-person and within-dyad processes.

We used the MLR estimation method, which is robust to non-normality and missing data, providing more reliable parameter estimates. Autoregressive paths for maternal and paternal depressive symptoms were systematically constrained and unconstrained, and Satorra–Bentler difference tests were used to determine whether these paths should be freely estimated or constrained (Satorra & Bentler, 1994). The following fit statistics were used to evaluate model fit: chi-square (*p* > .05 excellent), comparative fit index (CFI; > 0.90 acceptable, > 0.95 excellent), root mean square error of approximation (RMSEA; < 0.08 acceptable, < 0.05 excellent), and the standardized root mean square residual (SRMR; < 0.08 acceptable, < 0.05 excellent) (Hu & Bentler, 1999).

## Results

### ICC

The ICCs for both mothers (0.54, 95% CI: [0.47, 0.62]) and fathers (0.50, 95% CI: [0.42, 0.58]) indicate a moderate level of stability in depressive symptoms over time. These values indicate that approximately half of the variance in depressive symptoms is attributable to between-person differences (i.e., variance explained by individual characteristics, such as personality, depression history, or coping skills) and half is attributable to within-person fluctuations (i.e., variance explained by change over time due to situational factors, such as stress, life events, or parenting challenges). Given these ICC values, the RI-CLPM is well-suited for this analysis.

### Model fit

Satorra–Bentler scaled difference tests indicated that constraining the autoregressive paths for depressive symptoms to be equal across time resulted in a statistically significant decrement in model fit for both maternal (Δχ^2^(3) = 40.64, *p* < .001) and paternal depressive symptoms (Δ*χ*^2^(3) = 41.18, *p* = .001). Therefore, the autoregressive paths were not constrained to be equal for maternal or paternal depressive symptoms. The final model demonstrated good model fit, *χ*^2^(21) = 21.80, *p* = .411, TLI = 0.99, CFI = 1.00, RMSEA = 0.02, 90% CI [0.00, 0.08], and SRMR = 0.06. We present all model estimates in Table 2.

### Within-person variance

**Figure 1.**
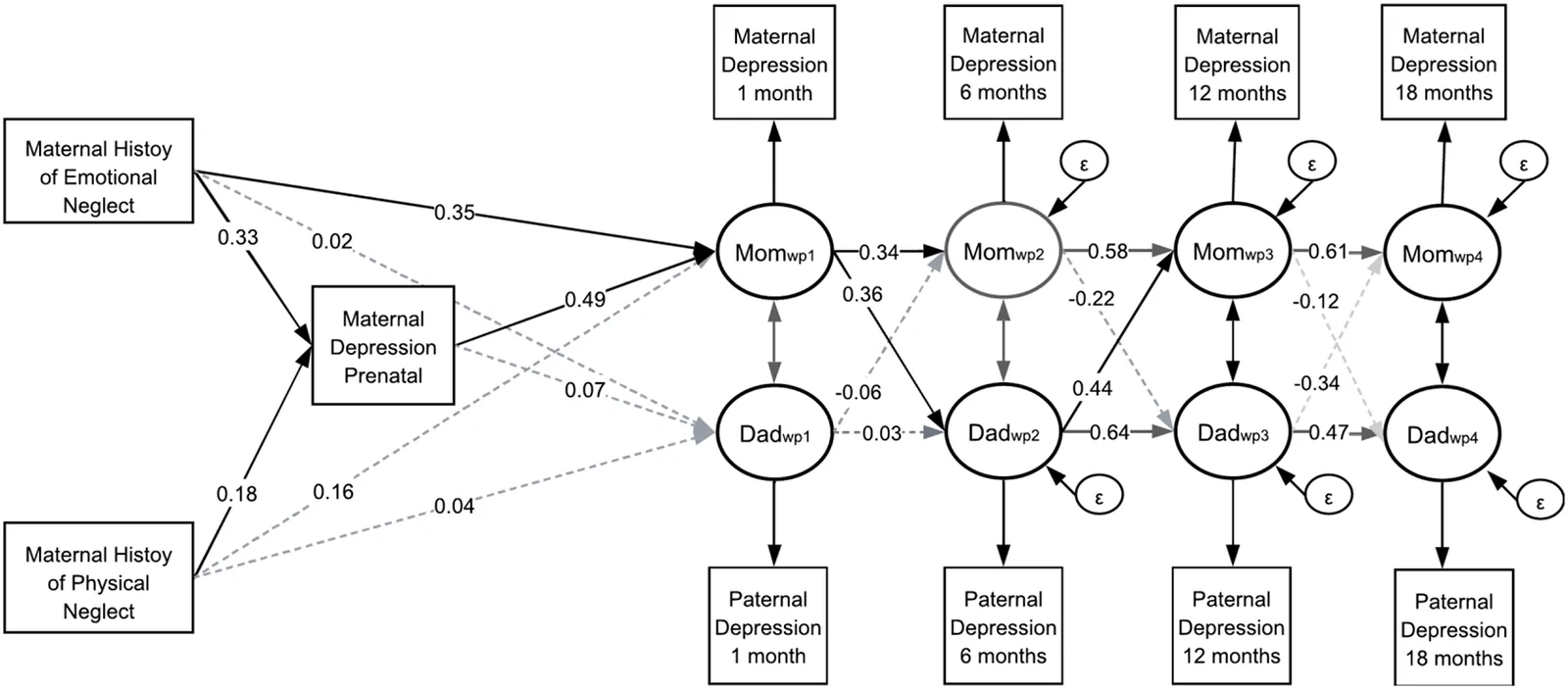
Random Intercept Cross-Lagged Panel Model Results **Note**. Standardized coefficients (β) are presented. Solid lines indicate statistically significant paths (*p* < .05), whereas dotted lines indicate nonsignificant paths. “wp” denotes within-person latent deviations (i.e., time-specific fluctuations around each individual’s expected level after accounting for stable differences). Between-person random intercept factors were included in the estimated RI-CLPM but are omitted from the figure for visual clarity; see Table 2. Within-wave covariances between maternal and paternal within-person residuals were freely estimated.

As indicated in Figure 1, all autoregressive paths for maternal depressive symptoms were statistically significant, indicating stability in within-person deviations in symptoms over time. Specifically, maternal depressive symptoms at 1 month postpartum were positively associated with symptoms at 6 months, with a medium effect size (*β* = 0.34). Symptoms at 6 months were positively associated with symptoms at 12 months (*β* = 0.58) and symptoms at 12 months were positively associated with symptoms at 18 months (*β* = 0.61), both with large effect sizes.

In contrast, not all autoregressive paths for paternal depressive symptoms were statistically significant. Specifically, within-person deviations in paternal depressive symptoms at 1 month postpartum were not significantly associated with within-person deviations at 6 months postpartum (*β* = 0.03). However, subsequent autoregressive paths were statistically significant, indicating that within-person deviations in paternal depressive symptoms at 6 months were positively associated with deviations at 12 months, with a large effect size (*β* = 0.64), and deviations at 12 months were positively associated with deviations at 18 months, with a medium effect size (*β* = 0.47).

With respect to associations between maternal and paternal depressive symptoms over time, maternal depressive symptoms at 1 month were statistically significantly positively associated with paternal depressive symptoms at 6 months, with a medium effect size (*β* = 0.36; see Figure 1), after accounting for paternal depressive symptoms at 1 month. In turn, paternal depressive symptoms at 6 months were statistically significantly positively associated with maternal depressive symptoms at 12 months, also with a medium effect size (*β* = 0.44), after accounting for maternal depressive symptoms at 6 months. No other cross-lagged paths were statistically significant (see Table 2). Concurrent within-wave associations between maternal and paternal depressive symptoms were freely estimated at each postpartum assessment and are reported in Table 2. None of these concurrent associations were statistically significant, suggesting limited evidence for shared time-specific influences beyond lagged spillover effects in this sample.

### Between-person variance

On average across time, maternal depressive symptoms were statistically significantly positively correlated with paternal depressive symptoms (*β* = 0.52, 95% CI [0.06, 0.99], *SE* = 0.24, *p* = .026), suggesting that mothers with generally higher depressive symptoms tended to have partners with generally higher depressive symptoms.

### Maternal prenatal depressive symptoms

Maternal prenatal depressive symptoms were statistically significantly positively associated with mothers’ 1 month postpartum within-person deviations in depressive symptoms (*β* = 0.49), as well as with higher stable (between-person) maternal depressive symptom levels across the postpartum period (*β* = 0.87), with medium to large effect sizes. In contrast, maternal prenatal depressive symptoms were not significantly associated with fathers’ 1 month postpartum within-person deviations in depressive symptoms or their stable (between-person) depressive symptom levels.

### Maternal history of neglect

More severe maternal childhood emotional and physical neglect were each statistically significantly positively associated with higher maternal depressive symptoms during pregnancy, with medium (*β* = 0.33) and small effect sizes (*β* = 0.18), respectively. In addition, greater maternal emotional neglect was statistically significantly positively associated with greater within-person deviations in maternal depressive symptoms at 1 month postpartum (*β* = 0.35; see Figure 1) but was not associated with fathers’ within-person deviations in depressive symptoms at 1 month. Maternal-reported physical neglect was not significantly associated with either maternal or paternal within-person deviations in depressive symptoms at 1 month postpartum. Neither emotional nor physical neglect was significantly associated with stable (between-person) levels of maternal or paternal depressive symptoms across the postpartum period (see Table 2).

### LCM-SR model

A LCM-SR was estimated to evaluate the presence of systematic between-person variability in depressive symptom trajectories across the postpartum period. The model failed to converge within the maximum number of iterations and produced improper solutions, including negative residual variances for later time points. These findings suggest that there was insufficient information to estimate reliable slope variability in the present sample and support the use of the RI-CLPM for examining time-specific within-person and within-dyad processes.

## Discussion

The goal of this study was to examine stability in, and bidirectional associations between, maternal and paternal depressive symptoms from 1 to 18 months postpartum. We also examined whether maternal history of childhood neglect and maternal prenatal depressive symptoms contributed to early perinatal risk. Four key findings emerged. First, maternal history of childhood neglect was associated with elevated maternal depressive symptoms during pregnancy and emotional neglect was associated with maternal depressive symptoms at 1 month. Second, maternal prenatal depressive symptoms were significantly associated with elevated maternal depressive symptoms at 1 month postpartum, including time-specific elevations relative to mothers’ typical symptom levels; they were not associated with paternal depressive symptoms. Prenatal symptoms were also associated with stable between-person differences in maternal, but not paternal, depressive symptoms across the postpartum period. Third, maternal depressive symptoms showed strong autoregressive stability across all postpartum assessments, while paternal depressive symptoms were less stable early in the postpartum period, with significant stability emerging only later. Fourth, cross-lagged analyses revealed bidirectional within-dyad associations over time. Higher maternal depressive symptoms at 1 month postpartum predicted subsequent within-person increases in paternal depressive symptoms at 6 months, and higher paternal depressive symptoms at 6 months subsequently predicted within-person increases in maternal depressive symptoms at 12 months. Together, these findings advance understanding of PPD by highlighting the importance of maternal childhood neglect, the central role of prenatal depressive symptoms in shaping early postpartum adjustment, and the dynamic, reciprocal nature of depressive symptoms within couples across the postpartum period.

### Maternal history of neglect

Our findings indicate that maternal childhood emotional and physical neglect play a meaningful role in shaping vulnerability to depressive symptoms during the earliest stages of the perinatal period. Both emotional and physical neglect were associated with higher prenatal maternal depressive symptoms, suggesting that neglect-related vulnerabilities may become especially salient during pregnancy, a developmental transition marked by profound biological, psychological, and relational change. This pattern is consistent with prior work indicating that pregnancy may function as a sensitive period during which early caregiving experiences are reactivated (Ayers et al., 2019; Hutchens et al., 2017). Pregnancy involves substantial physiological reorganization, including changes in inflammatory functioning, HPA axis activity, and stress responsivity, alongside heightened emotional and relational demands (Glynn & Sandman, 2011; Christian, 2015). Individuals with histories of childhood neglect may be particularly vulnerable during this period because neglect is associated with long-term alterations in stress regulation, emotion processing, and perceived social support (Heleniak et al., 2016; Khoury et al., 2021). Together, these findings suggest that neglect-related vulnerabilities may first emerge or become especially pronounced during pregnancy.

In contrast, emotional neglect was associated with greater early postpartum elevations in maternal depressive symptoms at 1 month. Emotional neglect is theorized to disrupt the development of healthy emotion functioning, including emotion awareness, regulation, and interpersonal support-seeking (Kim & Cicchetti, 2010; Shipman et al., 2007). These difficulties may be especially consequential in the early postpartum period, which places intense demands on emotional attunement, stress tolerance, and relational functioning. Consistent with this interpretation, emotional neglect has been linked to depressive symptoms and interpersonal vulnerabilities, including reduced perceived social support and negative self-referential processes, which may be especially relevant during the early postpartum period (Cohen et al., 2019; Jopling et al., 2020; Qin et al., 2021). In contrast, physical neglect, often reflecting material deprivation and inconsistent caregiving, may confer risk through more diffuse or longer-term biological pathways that are less tightly coupled to immediate postpartum emotional demands.

Notably, neither emotional nor physical neglect was associated with stable, between-person levels of maternal depressive symptoms across the postpartum period, indicating that neglect-related risk was concentrated in the early perinatal window rather than reflecting enduring trait-like differences in depression severity. Consistent with hypotheses, maternal neglect was not directly associated with paternal depressive symptoms at 1 month postpartum, nor with stable between-person differences in paternal depression. This pattern suggests that maternal early caregiving adversity does not independently shape fathers’ early postpartum mental health, but may instead exert indirect effects through maternal depressive symptoms, relationship strain, or coparenting stress. Such an interpretation aligns with family systems and stress spillover models, which emphasize that distal individual vulnerabilities influence partners primarily through proximal relational processes (Almeida et al., 1999; Williams & Cheadle, 2015).

Taken together, these findings support a developmentally timed risk framework in which maternal childhood neglect confers vulnerability that emerges during pregnancy and extends into the immediate postpartum period, rather than exerting broad, stable effects across time. This pattern underscores pregnancy and early postpartum as critical windows for identifying and supporting mothers with histories of emotional neglect. Screening for neglect during pregnancy may facilitate early prevention efforts before depressive symptoms become entrenched, potentially reducing downstream stress on partners and the broader family system (Giles & Khoury, 2025; O’Hara & Wisner, 2014).

### Maternal prenatal depressive symptoms

Maternal prenatal depressive symptoms were associated with greater within-person elevations in maternal depressive symptoms at 1 month postpartum and with higher stable levels of maternal depressive symptoms across the postpartum period. This pattern is consistent with extensive prior work demonstrating that PPD often represents a continuation of symptoms that emerge during pregnancy rather than a distinct, postpartum-onset condition (O’Hara & Wisner, 2014; Underwood et al., 2016). Pregnancy may therefore serve as a critical window during which vulnerability to persistent PPD becomes evident. Notably, prenatal depressive symptoms were not directly associated with fathers’ depressive symptoms at 1 month postpartum or with stable paternal depressive symptom levels. This finding suggests that prenatal maternal risk does not immediately translate into dyadic spillover but instead may operate indirectly through maternal postpartum symptoms. Together, these findings reinforce the importance of identifying and addressing depressive symptoms during pregnancy, not only to support maternal well-being but also to potentially prevent downstream interparental cascades that unfold later in the postpartum period.

### RI-CLPM stability paths

Our findings also revealed statistically significant autoregressive paths for maternal depressive symptoms from 1 through 18 months postpartum, indicating strong within-person stability in depressive symptom deviations across the postpartum period. Specifically, symptoms showed moderate continuity from 1 to 6 months (*β* = 0.34), but stability nearly doubled from 6 to 12 months (*β* = 0.58) and remained high from 12 to 18 months (*β* = 0.61). This pattern suggests that depressive symptoms may fluctuate more in the early postpartum period before becoming increasingly stable. These findings are broadly consistent with Clifford et al., 2024, who examined maternal and paternal depressive symptoms annually from 6 to 54 months postpartum and found that maternal depression persisted across the 4.5-year period, with mothers who experienced higher-than-usual symptoms at one time point being more likely to continue experiencing elevated symptoms a year later. However, Clifford et al. (2024) constrained maternal depressive symptoms to be stable across time in their model, whereas our results indicate that stability in maternal depressive symptoms is not constant but instead strengthens over the first 18 months. Our findings also align with broader evidence suggesting that maternal depression, particularly when present in the early postpartum months, often follows a chronic or recurrent course rather than resolving spontaneously (Jacques et al., 2020; Torres et al., 2019; Vliegen et al., 2014). This pattern highlights the urgency of early detection and treatment, as symptoms appear more malleable in the first 6 months but risk consolidating into chronic depression if left unaddressed.

For paternal depressive symptoms, we found less consistency in symptom stability compared to maternal symptoms. Specifically, paternal depressive symptoms at 1 month postpartum did not significantly explain differences in symptoms at 6 months. However, symptoms showed emerging stability from 6 to 12 months and remained stable, though with slightly smaller effect sizes, from 12 to 18 months. These results indicate greater stability in paternal depressive symptoms than was identified by Clifford et al. (2024), who found only one statistically significant autoregressive interval for paternal depressive symptoms across their study period. This difference may indicate that depressive symptoms in fathers follow a different developmental course in the first 18 months compared to later postpartum periods. These findings contribute to a growing body of literature indicating that paternal PPD follows a distinct pattern from maternal depression. Previous research suggests that fathers often experience a delayed onset of depressive symptoms, influenced by factors such as relationship strain, financial stress, and lack of social support (Cameron et al., 2016; Paulson & Bazemore, 2010). Our results are consistent with this pattern, as the stability of paternal symptoms appears to emerge later in the postpartum period, in contrast to maternal symptoms, which show strong stability from the earliest time points.

One possible explanation is that fathers initially experience transient mood disturbances due to adjustment difficulties, sleep deprivation, or shifting family dynamics. However, these symptoms may not persist unless reinforced by ongoing stressors, coparenting challenges, or cumulative family strain. This aligns with family systems theory (Belsky & Rovine, 1990; Cox & Paley, 1997) and stress spillover models (Almeida et al., 1999), which suggest that early situational distress can escalate into chronic symptoms if stress accumulates within the family system. Additionally, adjustment to parenthood models highlight how changes in roles and identities can cause temporary distress that may stabilize or worsen depending on the presence of supportive resources (Doss et al., 2009; Seyed Karimi et al., 2021). Thus, just as maternal symptoms require early detection, paternal depression may call for continued monitoring and intervention in the later postpartum months, when symptoms shift from being transient to more stable.

### RI-CLPM bidirectional effects

Our analyses revealed bidirectional associations between maternal and paternal depressive symptoms that were time-specific rather than uniform across the postpartum period. Maternal depressive symptoms at 1 month postpartum predicted subsequent increases in paternal depressive symptoms relative to fathers’ typical levels at 6 months, whereas paternal depressive symptoms at 6 months predicted subsequent increases in maternal depressive symptoms relative to mothers’ typical levels at 12 months. Other cross-lagged paths were small or nonsignificant, suggesting that dyadic influence is not constant but emerges during particular developmental windows.

This time-specific pattern is consistent with family systems and stress spillover models, which posit that emotional influence between partners varies as family roles and caregiving demands shift across the postpartum period (Almeida et al., 1999; Cox & Paley, 1997). During the early postpartum interval (1 to 6 months), mothers typically assume a disproportionate share of caregiving responsibilities and experience substantial biological, emotional, and role-related changes. Heightened maternal distress during this period may therefore be especially likely to spill over to partners through increased household tension, disrupted coparenting, and reduced emotional availability, which may explain why maternal symptoms at 1 month forecast paternal symptoms at 6 months. Notably, mothers in the present sample exhibited the highest rates of clinically elevated depressive symptoms at the one-month postpartum assessment. This concentration of maternal distress during the early postpartum period may have increased the salience of maternal symptoms within the family system and contributed to their subsequent association with paternal depressive symptoms.

In contrast, the later postpartum interval (6 to 12 months) may reflect a shift in family dynamics in which paternal symptoms exert greater influence. Around six months postpartum, infants become more developmentally demanding, many mothers return to work, and coparenting roles and expectations begin to stabilize. Paternal depressive symptoms during this period may therefore disrupt dyadic functioning and increase maternal emotional burden, consistent with stress spillover frameworks. The emergence of a paternal-to-maternal pathway, alongside the absence of a reciprocal maternal-to-paternal effect during this interval, suggests a developmentally contingent asymmetry in influence as family roles evolve.

This pattern differs from prior work that identified only unidirectional effects (e.g., Clifford et al., 2024), potentially due to differences in assessment timing. Whereas earlier studies examined longer intervals beginning later in the postpartum period, the inclusion of earlier assessments in the present study may have allowed detection of more immediate, reciprocal influences closer to the transition to parenthood. Taken together, these findings suggest that dyadic influences on depressive symptoms emerge during discrete developmental windows rather than operating uniformly over time, highlighting the importance of timing when designing screening and intervention efforts. Traditionally, maternal mental health has been considered the primary driver of family emotional climate, with less emphasis on how paternal well-being affects maternal adjustment (Paulson & Bazemore, 2010; Paulson et al., 2016). However, our results suggest that fathers’ mental health difficulties may contribute to worsening maternal symptoms, potentially creating a cycle of distress that affects both parents. Importantly, dyadic spillover effects between parents’ depressive symptoms likely operate in concert with infant-related factors that change across the postpartum period. Child characteristics such as prematurity, early sleep and regulatory difficulties, or infant biological sex may alter parental stress exposure at specific developmental stages, thereby shaping the timing and strength of reciprocal symptom associations between parents. Importantly, although maternal and paternal depressive symptoms differed in stability and timing of cross-lagged associations across the postpartum period, these findings should not be interpreted as evidence of distinct developmental trajectories. The RI-CLPM separates stable between-person differences from within-person fluctuations and reciprocal associations, rather than estimating growth parameters. Thus, our results reflect differences in within-person stability and dyadic spillover, not formally modeled trajectories. Maternal symptoms showed stronger early within-person stability, whereas paternal symptoms showed weaker early stability, with stronger associations emerging later, a pattern consistent with prior work but not a direct test of differential developmental trajectories, which would require growth modeling.

The timing specificity of these findings has clear clinical implications. First, results suggest that perinatal mental health screening may benefit from a dyadic and repeated approach, rather than relying on single time-point, mother-focused assessments. Maternal depressive symptoms early in the postpartum period were most predictive of later paternal symptoms, indicating that identifying and supporting mothers during pregnancy and the first months postpartum may have downstream protective effects for partners. Conversely, paternal depressive symptoms around 6 months postpartum emerged as a salient predictor of later maternal symptoms, suggesting that this period may be a critical window for monitoring fathers’ mental health, an aspect of perinatal care that remains underemphasized in many clinical settings. Screening only one parent, or screening only early in the postpartum period, may therefore underestimate family-level risk and miss opportunities for prevention before symptoms consolidate.

Second, the observed bidirectional and time-limited spillover effects underscore the potential value of dyadic or family-based interventions that explicitly address interparental processes, coparenting stress, and emotional spillover. Early postpartum interventions may be most effective when focused on stabilizing maternal mood and reducing early family strain, whereas later interventions may benefit from increased attention to paternal mental health as caregiving responsibilities expand and coparenting roles solidify. Tailoring intervention timing and targets to these shifting developmental phases may help interrupt cascading symptom trajectories within couples and reduce the likelihood that transient distress evolves into chronic depression for either parent. Together, these findings support developmentally informed, dyadic approaches to perinatal mental health that better capture how parental symptoms unfold within family systems across the first year postpartum.

### Limitations

Several limitations should be noted. Our sample, drawn from a single southeastern U.S. metropolitan area, was predominantly middle-class and non-Hispanic White, which may limit generalizability to other contexts. Examining dyadic symptom processes in families with greater racial, ethnic, socioeconomic, and cultural variability would allow assessment of whether timing-specific spillover effects reflect universal features of the perinatal transition or are shaped by contextual stress exposure and available supports. In addition, we did not examine contextual moderators such as social support, relationship quality, or caregiving burden, which may condition the strength or direction of within-dyad spillover effects. Future work testing moderation would help identify for whom and under what circumstances dyadic symptom transmission is most pronounced. Second, while the majority of our study participants had depressive symptoms in the non-clinical range, 36% of mothers and 18% of fathers reported scores above the clinical cutoff at one or more assessments across the perinatal period. As such, the findings from this sample may not generalize to higher-risk samples. However, understanding dynamics across the continuum of depression severity is important, as subthreshold depressive symptoms are associated with meaningful functional impairment (An et al., 2022; Hare et al., 2021; Rodríguez et al., 2012).

Third, prenatal depressive symptoms and childhood neglect were available only for mothers. The absence of paternal childhood adversity data and prenatal depression data limits our ability to examine parallel pathways or to test whether similar mechanisms operate for fathers. Including paternal histories of neglect and prenatal depressive symptoms in future studies would provide a more comprehensive understanding of how each parent’s developmental background and perinatal emotional functioning influence their own and their partner’s adjustment to parenthood. Fourth, our use of 6 month measurement intervals may have missed shorter-term fluctuations in depressive symptoms that are common in the postpartum period. More intensive longitudinal study designs, such as those facilitated by ecological momentary assessment, would allow future studies to capture rapid, day-to-day fluctuations in parental mood and to apply dynamic modeling approaches (e.g., dynamic structural equation modeling) that are specifically suited to such data.

Fifth, the study relied exclusively on self-report measures of depression. Incorporating multi-method approaches, such as clinical interviews or observational assessments, would strengthen future research by reducing shared method variance and providing a more nuanced picture of parent mental health. Sixth, as all dyads in the present study were heterosexual couples, extending this work to non-heteronormative family structures would help distinguish processes driven by gendered caregiving roles from those reflecting broader dyadic dynamics. Seventh, because 91% of mothers were married or in a domestic partnership, our sample primarily reflects stable, cohabiting couples. Dyadic spillover patterns may differ in families experiencing relationship instability or non-cohabiting arrangements. Finally, participation of fathers was optional in the larger study, contributing to reduced paternal participation relative to mothers, and ultimately a smaller dyadic analytic sample. Although families included in dyadic analyses did not differ on key sociodemographic or baseline variables from those without sufficient data to be included, differential participation may still limit representativeness and should be considered in future recruitment strategies.

### Conclusion

This study is among the first to examine within-person, bidirectional associations between maternal and paternal depressive symptoms across the first 18 months postpartum using a RI-CLPM. By assessing depressive symptoms at 1, 6, 12, and 18 months postpartum, we were able to distinguish stable between-person differences from time-specific within-person fluctuations and to identify how changes in one parent’s symptoms predicted subsequent changes in the other parent’s symptoms over time. Our findings underscore the importance of monitoring both parents’ mental health across the postpartum period. Maternal depressive symptoms showed strong stability beginning early postpartum, whereas paternal depressive symptoms were not stable from 1 to 6 months but demonstrated increasing stability from 6 to 18 months. These patterns suggest that maternal symptoms may become stable earlier, while paternal symptoms may emerge and stabilize later, highlighting the importance of continued screening and support for both parents beyond the immediate postpartum period.

Consistent with a family systems framework, our cross-lagged analyses revealed reciprocal associations between maternal and paternal depressive symptoms, indicating that parental mental health is dynamically interconnected. Maternal symptoms in the early postpartum period were associated with later paternal symptoms, which in turn predicted subsequent maternal symptoms, suggesting the potential for cyclical, within-dyad processes that unfold over time. Clinically, these findings support the value of integrated, dyadic approaches to perinatal mental health screening and intervention rather than focusing on one parent in isolation. Finally, our results highlight the role of maternal developmental history in shaping early perinatal risk. Maternal childhood emotional and physical neglect were associated with higher prenatal depressive symptoms, and emotional neglect uniquely predicted higher maternal depressive symptoms at 1 month postpartum. Importantly, maternal neglect did not directly predict paternal depressive symptoms, suggesting that early caregiving adversity may influence family functioning indirectly through its association with maternal perinatal mental health. Together, these findings emphasize pregnancy and the early postpartum period as critical windows for identifying mothers with histories of emotional neglect and elevated depressive symptoms, offering opportunities for early intervention that may benefit both parents and the broader family system.

## Figures and Tables

**Table 1.** Demographics

**Mother’s age (M, SD)**	31.15 (4.30)
**Father’s age (M, SD)**	32.74 (5.54)
**Marital status**	
Married or domestic partnership	91%
Single, never married	6%
Divorced	2%
Separated	1%
**Mother’s ethnicity, % non-Hispanic**	93%
**Father’s ethnicity, % non-Hispanic**	94%
**Mother’s race**	
White	89%
Other race	4%
Asian	3%
Black or African American	2%
Native Hawaiian or Other Pacific Islander	2%
American Indian or Alaska Native	0%
**Father’s race**	
White	90%
Other race	3%
Asian	4%
Black or African American	3%
Native Hawaiian or Other Pacific Islander	0%
American Indian or Alaska Native	0%
**Mother’s education**	
High school graduate or equivalent (i.e. GED)	0%
Associate degree	4%
Bachelor's degree	44%
Some college credit, no degree	7%
Graduate degree	39%
Trade/technical/vocational training	3%
Other	0%
Did not answer	3%
**Father’s education**	
High school graduate or equivalent (i.e. GED)	0%
Associate degree	4%
Bachelor's degree	3%
Some college credit, no degree	11%
Graduate degree	49%
Trade/technical/vocational training	10%
Other	21%
Did not answer	2%
**Father’s Relationship with child**	
Biological father	98%
Other	1%
Did not answer	1%
**Annual household income**	
<$60,001	22%
$60,001–90,000	25%
$90,001–150,000	32%
Greater than $150,000	20%
**First time parent (for mothers)**	64%

Note. All demographic data presented is from the initial assessment during pregnancy.

**Table 2.** Random Intercept Cross-Lagged Panel Model Results

	*β*	95% CI Lower	95% CI Upper	*p*
**Effects of Maternal Neglect Depression on Maternal Prenatal Depressive Symptoms**				
Emotional Neglect → Mom prenatal	0.33	0.11	0.55	.003
Physical Neglect → Mom prenatal	0.18	0.02	0.34	.032
**Effects of Maternal Prenatal Depression on Depressive Symptoms at T1**				
Emotional Neglect → Mom T1	0.35	0.04	0.67	.029
Physical Neglect → Mom T1	0.16	−0.20	0.51	.390
Emotional Neglect → Dad T1	0.02	−0.35	0.39	.912
Physical Neglect → Dad T1	0.04	−0.16	0.24	.710
**Maternal Prenatal Depression on Within-Person Parental Depressive Symptoms at T1**				
Mom prenatal → Mom T1	0.49	0.12	0.89	.014
Mom prenatal → Dad T1	0.07	−0.23	0.37	.650
**Autoregressive and Cross-Lagged Within-Person Effects (T1 → T2)**				
Mom T1 → Mom T2	0.34	0.08	0.60	.010
Dad T1 → Mom T2	−0.06	−0.33	0.20	.648
Mom T1 → Dad T2	0.36	0.05	0.68	.024
Dad T1 → Dad T2	0.03	−0.52	0.26	.892
**Autoregressive and Cross-Lagged Within-Person Effects (T2 → T3)**				
Mom T2 → Mom T3	0.58	0.31	0.84	<.001
Dad T2 → Mom T3	0.44	0.06	0.81	.022
Mom T2 → Dad T3	−0.22	−0.64	0.20	.309
Dad T2 → Dad T3	0.64	0.29	0.99	<.001
**Autoregressive and Cross-Lagged Within-Person Effects (T3 → T4)**				
Mom T3 → Mom T4	0.61	0.17	1.05	.006
Dad T3 → Mom T4	−0.34	−0.83	0.06	.081
Mom T3 → Dad T4	−0.12	−0.44	0.20	.462
Dad T3 → Dad T4	0.47	0.08	0.85	.018
**Time-Invariant Predictors of Between-Person Parental Depressive Symptoms**				
Emotional Neglect → Mom Between	0.26	−0.04	0.56	.090
Physical Neglect → Mom Between	0.25	−0.03	0.53	.075
Mom Prenatal → Mom Between	0.87	0.67	1.07	<.001
Emotional Neglect → Dad Between	0.09	−0.27	0.45	.631
Physical Neglect → Dad Between	0.01	−0.45	0.33	.933
Mom Prenatal → Dad Between	0.27	−0.05	0.59	.096
**Within-Wave Covariances Between Maternal and Paternal Residual Depressive Symptoms**				
Mom T1 with Dad T1	−0.20	−0.53	0.13	.243
Mom T2 with Dad T2	−0.14	−0.49	0.21	.428
Mom T3 with Dad T3	0.41	−0.22	1.04	.198
Mom T4 with Dad T4	−0.02	−0.32	0.28	.885

*Note*. Mom = mothers’ depressive symptoms; dad = fathers’ depressive symptoms; Mom prenatal = mother’s prenatal depressive symptoms; T1 = 1 month postpartum, T2 = 6 months postpartum, T3 = 12 months postpartum, T4 = 18 months postpartum, Paths labeled ‘**Mom Between’** and ‘**Dad Between’** represent associations between predictors and the between-person (random intercept) components of maternal and paternal depressive symptoms, respectively

## Data Availability

**Availability of Data:** The data necessary to reproduce the analyses presented in this study are publicly available via the Open Science Framework at: https://osf.io/x8wme/?view_only=2c9a9b31bc6b41a5802db4a1cab6aa8d **Availability of Code:** All analytic code used to conduct the analyses is publicly available via the Open Science Framework at the link provided above. **Availability of Methods/Material:** All study materials and documentation necessary to attempt replication of the findings are publicly available via the Open Science Framework at the link provided above. This study and analytic plan were not pre-registered. Data and code are available at https://osf.io/x8wme/overview?view_only=2c9a9b31bc6b41a5802db4a1cab6aa8d.
